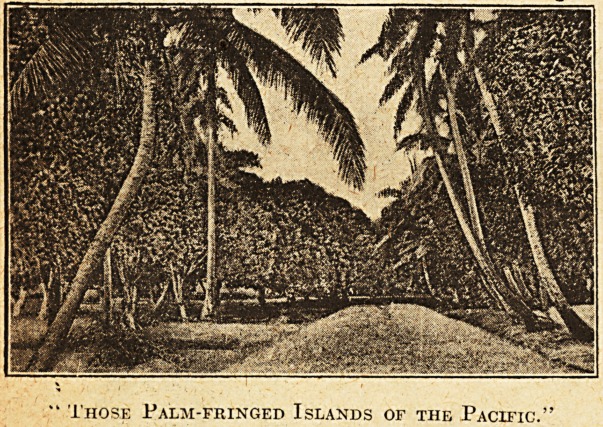# Curiosities of Native Treatment in Fiji

**Published:** 1919-02-22

**Authors:** T. R. St.-Johnston

**Affiliations:** Colonial Civil Service.


### CURIOSITIES OF NATIVE TREATMENT IN FIJI.

### III. Native Surgery, Pain-bearing Capacity and Customs.

By T. R. ST.-JOHNSTON, M.R.C.S., L.R.C.P., Colonial Civil Service.

A minor operation that the native surgeons were always very good at was that of circumcision, universally practised among them from remote ages—surely another connecting link with their Eastern origin! This was done with the split bamboo knife, dressings of "tappa" being used on the completion of the operation. Curiously enough it had two names, "Tevé" when spoken of among men, but a forbidden word in the presence of women, when "Kula" had to be used. There was a curious operation somewhat akin to this called "Cokalosi," which consisted of passing a grooved wooden staff down the urethra and slitting up for a distance of about three inches, thereby allowing "all the bad blood and evil" to run out. In certain instances it might have had the effect of the venesections of our grandfathers, but it was rather drastic.

A surgical remedy for rheumatism and vague muscular pains was the cutting of deep incisions with sharp shells generally across the back; a sort of counter-irritant that the natives still have much faith in. One often finds half-a-dozen of these big keloid scars across the smooth brown skin of some magnificent specimen of humanity, who has regarded the operation with perfect nonchalance. Questions of pain affect the natives in curiously different ways. Crushing wounds that would make a white man sick with agony they often do not seem to mind at all, though they may flinch from the scratch of a knife. I think that they have really a high degree of sensitiveness, and the *anticipation* of pain with them is the worst part. When it actually has arrived they can usually treat it with a stoical indifference.

When they work themselves up to a pitch of frenzy, as in the witchcraft practices of "Luveniwai" (not by any means extinct yet), they can endure most extraordinary sufferings. The chief performers in these rites are beaten on the abdomen with small clubs to a degree little short of rupturing internal viscera; while in another case I heard of, where a man was supposed to have been entered by a god, he chewed off portions of a burning firebrand, quivering the while in a veritable ecstasy.

These are instances where perhaps the sense of pain was subordinated to a self-produced brain storm, but for a cold-blooded submission to pain the case of the Chief of Moala Island stands out in my memory. This man had a large synovitis of the knee-joint, and when I visited his island on one of my periodical inspections I found that he had persuaded an old native surgeon to try one of their ancient remedies for this, and had had five deep holes burned through the living tissues into the substance of his patella with white-hot pointed stones, so that his knee resembled the pattern of a five of diamonds. Yet strange to say he received no ill results from this radical treatment, and the synovitis shortly afterwards disappeared, thus somewhat nullifying the effects of my harangue at his folly.

Speaking of burning, there is a curious ceremony performed by the natives of Beqa Island, which has never been explained. Certain tribes there have the gift of being able to walk barefooted on a large pit of white-hot stones, and continue to walk round and round in single file, chanting an ancient song as they do so. There is no trickery about this, and many whites have witnessed the performance, and made close inspections of the feet of the performers before and after the ceremony, while the stones have burned up handkerchiefs and other articles thrown on to them.

Not so insensitive to burning was a scatter-brained youth from the Sigatoka River district that I once had as a kitchen assistant. He came running in to me one day scared out of his life

A Native Medical Student. A Case of Elephantiasis.

and pointing to his mouth and throat, which were all scorched. I found out afterwards that he had taken some paraffin to encourage the kitchen fire, and not finding a handy receptacle to carry it from the tin in the store-room, he had sucked up a big mouthful of it, and then putting his lips to the bars had blown it into the fire, with the result that a stream of liquid flame had jumped back into his mouth!

### OBSTETRICS.

It is not so many generations since midwifery in England began to take its place as a science by the side of its more esteemed partners, medicine and surgery. And the ways of the midwife of old-time England hardly seem less strange in comparison with the science of the modern obstetrician than do the methods of a native "yalewa vuku," or midwife, out in the wilds of Fiji. Bearing this

Previous articles appeared in the "Hospital" of January 18, page 333, and February 8, page 405.

**Curiosities of Native Treatment in Fiji—(*cont.*).**

in mind, and always remembering that what may seem strange in one country is common usage in another, one can look with greater latitude on some of the curious performances that used to be, and sometimes still are, observed at the delivery of a child in those palm-fringed islands of the Pacific.

Firstly, the position of delivery is interesting in view of the controversy that is still waged in Europe as to the best way the mother should lie. In Fiji the woman is always placed in an upright sitting position, with her legs stretched out straight before her, and her body reclining back, supported by the arms of her friends. There is usually a little collection of womenfolk in the house, gathered in from neighbouring villages, for all the near relatives like to be present on these important occasions. The anxious father, just like his white brother, hovers round outside the house, ready to make himself useful, but generally in the way.

The midwife then approaches and makes a careful digital examination, and tells the bystanders what the presentation is, and then—as the pains get progressively more severe—the sitting woman is gradually lowered by her supporters into a re-

"Those Palm-fringed Islands of the Pacific."

clining position, though never allowed to be quite flat. The "yalewa vuku" are really quite skilled in these digital examinations, and it is customary in the early months for the expectant mother to be taken down to the river and there to be so examined, under water, by the midwife with a view to ascertaining if pregnancy has actually taken place or not, their theory being that the parts can be more easily manipulated under water. This is not such a risky performance as it might seem, since the river waters out there are naturally very much warmer than at home.

It may not be out of place here to mention the curious custom that they have of the "tobi." When a girl gets married, or is no longer a virgin, which means (if she has hitherto concealed her little amours) when she is at last pregnant, she is compelled by ancient custom to sacrifice the "tobi," a small cluster of about half-a-dozen finely plaited locks on one side of the head that all unmarried girls used to wear, and still do in many districts.

When the baby is at last born, generally without much trouble to the mother, the midwife takes it and waits a few moments, and then solemnly measures off the cord to the length of the infant's knee. Some one now hands her a knife of previously prepared split bamboo, and she places the cord at the measured part across a smooth block of polished wood (usually a "kali," or head-rest), and severs it. Owing to the length of the cord remaining there is usually very little hæmorrhage, and this is easily controlled by the bandages of "tappa" (native cloth) that are wrapped round it. The cord was never tied until recent years.

A drink of very cold water is now usually given to the mother to help in the expulsive efforts of the uterus, and the midwife then turns her attention to the baby. As a rule, owing to the fact of the comparatively easy births, the babies do not mark their entry into the world by that obstinate refusal to breathe that is often so alarming among more civilised infants, but if there is any sign of obstruction by mucus, etc., the midwife is very punctilious about clearing this out; and if the trouble still continues, a little juice of the candle-nut tree (*Aleurites triloba*) is forced down the throat to cause the baby to vomit, which it generally does, and simultaneously the reflex action set up causes the desired respirations to start. The baby is then carefully covered all over with yellow turmeric powder—an important part of the ceremony—and put aside in sheets of fine "tappa," the whole being placed upon a small mat of beautifully woven narrow grasses, bordered with scarlet parrots' feathers, though the latter have in these modern degenerate days given place to the more prosaic red wool.

Meanwhile the placenta has probably been expelled, and clots have begun to come away. One advantage of the semi-upright position is clearly seen here, as gravitation helps to empty the uterus. An unexpelled placenta is the one dread of the Fijian midwives, and only in certain parts of the country will they dare to risk manual interference. Luckily this is not often necessary, as the uterus seldom gets tired out by a difficult labour. I have often seen mothers in the Colo Mountains out and at work within two or three days of a confinement; and Thomson, an authority on Fijian customs, even mentions a case where a pregnant woman went out to work in the morning and returned in the evening with a baby in her arms and a load of firewood on her back!

As a rule when there is no other burden a Fijian mother carries the baby slung across her back in a cloth, and when it gets a little older it sits upright in a sort of sling, straddle-legged, across her back. The Indian immigrant, on the contrary, carries her child on her side, facing inwards, and astride one hip.

## Figures and Tables

**Figure f1:**
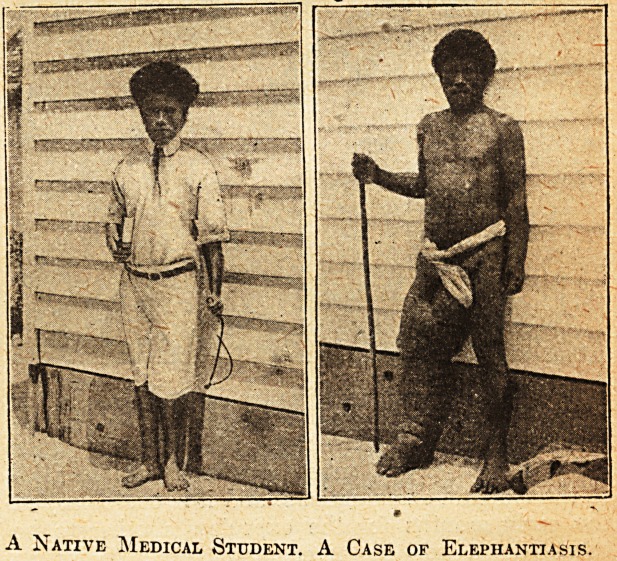


**Figure f2:**